# Solid Dose Form of Metformin with Ethyl Eicosapentaenoic Acid Does Not Improve Metformin Plasma Availability

**DOI:** 10.4236/pp.2016.71005

**Published:** 2016-01-14

**Authors:** Jeffrey H. Burton, William D. Johnson, Frank L. Greenway

**Affiliations:** Pennington Biomedical Research Center, Baton Rouge, USA

**Keywords:** AUC, Bioavailability, Eicosapentaenoic Acid, Metformin, Pharmacokinetics

## Abstract

**Background:**

The purpose of the study was to investigate effects of ethyl eicosapentaenoic acid on pharmacokinetics of metformin. Pharmacokinetic profiles of metformin and ethyl eicosapentaenoic acid when delivered separately or together in solid dose form were investigated and compared to determine whether the solid dose resulted in an altered metforminpharmacokinetics when given with or without food.

**Methods:**

A single-center, open-label, repeated dose study investigated the pharmacokinetic (PK) profile of metformin when administered in solid dose form with ethyl eicosapentaenoic acid compared to co-administration with icosapent ethyl, an ester of eicosapentaenoic acid and ethyl alcohol used to treat severe hypertriglyceridemia with metformin hydrochloride. Non-compartmental PK methods were used to compare area under the plasma concentration curve (AUC) and maximum plasma concentration (C_max_) between patients randomized to either the ester or separate medications group under both fasting and fed conditions.

**Results:**

Using these two PK parameters, results showed that metformin availability was higher under fasting conditions when delivered separately from icosapent ethyl. There were no group differences in the fed condition.

**Conclusions:**

The solid dose form of metformin and ethyl eicosapentaenoic acid did not improve the pharmacokinetics of metformin in terms of plasma availability, suggesting that little is to be gained over the separate administration of ethyl eicosapentaenoic acid and metformin hydrochloride.

## 1. Introduction

Metformin has been recommended as first line therapy for the treatment of type 2 diabetes due to its low cost, proven safety record, lack of weight gain and possible benefits for cardiovascular outcomes [1]. Type 2 diabetes is associated with triglyceride elevation, coronary heart disease and stroke [2]. The Nurse’s Health Study found an inverse association of fish intake and omega-3 fatty acids to cardiovascular death [3]. Women in the National Health and Nutrition Examination Survey (NHANES) who consumed fish more than once a week had half the age related risk of stroke compared to women who did not eat fish [4]. Based on this and other data, the American Heart Association recommended supplementary eicosapentaenoic acid for people who need to reduce their cardiovascular risk and are not successful through diet alone [5]. Icosapent ethyl is an ester of eicosapentaenoic acid (EPA) and ethyl alcohol. This ester is converted to its components in the body. Icosapent ethyl is approved by the FDA as a medication to reduce triglyceride levels in patients with severe hypertriglyceridemia over 500 mg/dL [6].

A metformin ester of the amino acid glycine has been created and tested in drug-naïve adults with type-2 diabetes. Metformin glycinate decreased haemoglobin A1c to a significantly greater degree than placebo [7]. Metformin glycinate also had a greater maximum concentration and area under the curve at equimolar doses compared to metformin hydrochloride [8] [9]. This observation suggested the possibility that the development of a single drug (metformin eicosapentaenoate) containing both metformin and EPA might also give greater metformin bioavailability. Metformin eicosapentaenoate would presumably not only treat the diabetes, but also decrease the elevation of triglycerides which are so often elevated in patients with diabetes. As described by the manufacturer, metformin eicosapentaenoate is an ionic salt that quickly separates into metformin base and eicosapentaenoate free fatty acid in an aqueous environment. It was hypothesized that, viathis solid dose form of delivery, the plasma availability of metformin would be improved and the gastrointestinal side effects would be reduced when compared to the separate co-administration of metformin and EPA. This study describes the pharmacokinetics of metformin when given with icosapent ethyl compared to the pharmacokinetics of metformin eicosapentaenoate in a fasted and fed state.

## 2. Methods

### 2.1. Study Design

Sixteen subjects were enrolled in the trial and were randomized 5:3 to either metformin eicosapentaenoateor to metformin plus icosapent ethyl, respectively. To be included, participants in the study had to be men or women (sterile, >1 year menopausal, or practicing adequate contraception) aged 18 to 65 years with: no history of chronic diseases, a BMI ≤ 30 kg/m^2^, no significant medical history including diabetes, hypertension or hyperlipidemia, negative urine alcohol and drug screening, and no usage of metformin or omega-3 fatty acid products within 2 months.

The first dose of the assigned drug therapy was given on day 1 of the study in the clinic after fasting for 10 – 12 hours. Plasma samples for PK analysis were taken at the time of drug consumption and at 0.5, 1, 2, 4, 8, and 12 hours following administration. Participants returned to the clinic 7 days later and were given the same dosage following a standardized meal. Again, plasma samples were drawn in an identical manner for subsequent PK analysis.

Metformin PK profiles were examined in a single-center, open-label, repeated dose study in which metformin was administered with icosapent ethyl or as metformin eicosapentaenoate in fasted and fed states. Metformin was delivered under the brand name Glucophage^®^ and icosapent ethyl under the brand name Vascepa^®^. The dosages for each were as follows: metformin eicosapentaenoate1500 mg (via four 375 mg capsules), metformin 500 mg (via one 500 mg tablet), and icosapent ethyl 1000 mg (via one 1000 mg gel cap).

### 2.2. Pharmacokinetic Parameters

The pharmacokinetic (PK) analysis of metformin used non-compartmental methodologies. Specifically, three measures were estimated from the data: area under the PK curve (AUC), maximum plasma concentration (C_max_) and time of maximum plasma concentration (t_max_). These measures were used to compare the processing of metformin when administered as metformin eicosapentaenoate or separately with icosapent ethyl. AUC was calculated using the trapezoidal rule. The notation AUC_last_ will be used to indicate that AUC was estimated only up to the last observed concentration at 12 hours post-administration and not extrapolated to infinity.

### 2.3. Statistical Analysis

The primary aim of the research described here was to compare PK profiles of metformin under fasting and fed conditions when administered as metformin eicosapentaenoate versus separately as metformin and icosapent ethyl. The profiles were compared via non-compartmental PK parameters as area under the curve AUC_last_ and C_max_. The analysis consisted of fitting two linear mixed effects models, each using one of the primary outcomes as the response variable. The models both contained fixed effects for treatment group, study condition (fasting/fed), and the interaction of these two variables. In addition, a random participant effect was included to account for within-subject correlation between repeated measurements taken under the fasting and fed conditions. Least squares means (LSM) were obtained from the models in order to compare the effects of the drug treatment groups under each study condition and to compare the effects of the study conditions within each treatment group. LSM were compared using two-sample t-tests. Since this study was exploratory in nature, two-sided t-tests were performed for each pair-wise comparison. All analyses were carried out using SAS/STAT^®^ software, Version 9.4 of the SAS System for Windows (Cary, NC, USA), and all tests were evaluated using significance level *α* = 0.05. A result was considered statistically significant if *p < α*.

## 3. Results

**Figure 1:**
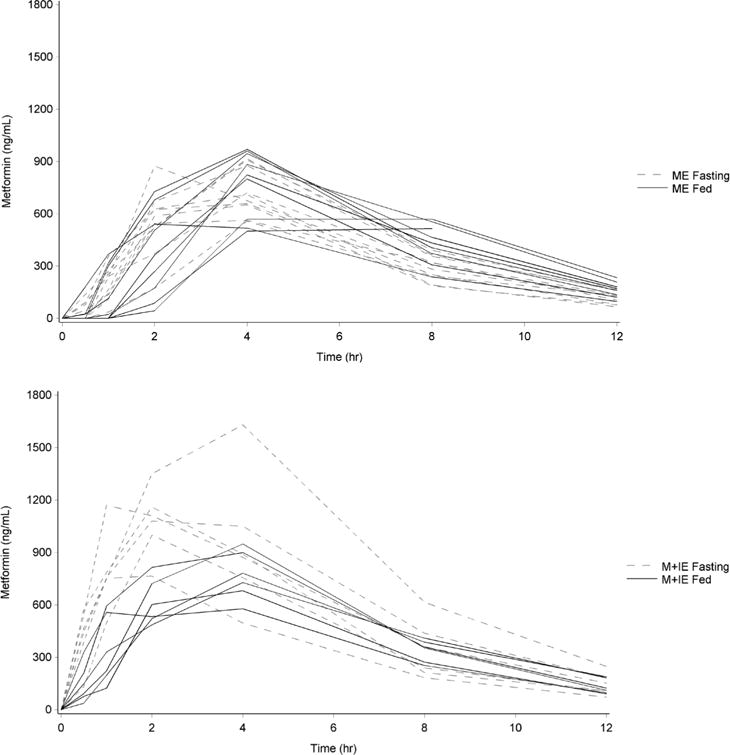
Individual metformin plasma concentration curves under each drug group and study condition; ME = metformin eicosapentaenoate, M + IE = metformin plus icosapent ethyl.

See Figure 1 for plots of individual metformin concentrations for participants in both drug treatment groups. For all analyses described here, plasma concentrations below the lower limit of quantification (BLQ) were treated as zero. Additionally, a participant missing any blood draw data at a given study visit had their complete data from that study visit excluded from the analysis. This was the case for two participants in the metformin eicosapentaenoate group at the second study visit.

**Figure 2:**
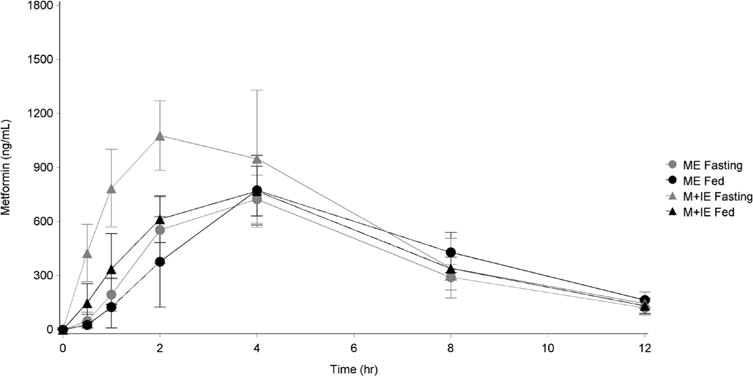
Group metformin plasma concenrations under each study condition (Mean ± SD); ME = metformin eicosapentaenoate, M + IE = metformin plus icosapent ethyl.

Summary measures of metformin plasma concentrations for each blood draw under both study conditions (fasting and fed) are summarized by treatment group in Table 1 and presented visually in Figure 2. Summary statistics of the PK parameters are presented in Table 2.

The specific comparisons that were carried out and the results are summarized in Table 3. The ratio of LSM is presented as a percentage along with 90% confidence intervals and *p*-values. The ratios are used to show relative differences rather than absolute; however, the associated *p*-values are from tests of the differences of LSM. For the calculation of each ratio, the numerator was the test group mean and the denominator was the reference group mean. Thus, a ratio greater than 100 indicates a larger mean in the test group.

The results comparing AUC_last_ demonstrated that the LSM of AUC_last_ for metformin under metformin eicosapentaenoate was lower than that for metformin in the metformin plus icosapent ethyl group under both fasting and fed conditions. This difference was only statistically significant, however, in the fasting condition (*p* = 0.001). This indicates that the average plasma concentration of metformin over the 12 hour period was significantly lower in subjects taking metformin eicosapentaenoate when the drugs were administered without food. The same result was observed for C_max_ in the fasting condition. The C_max_ of metformin under metformin eicosapentaenoate while fasting was significantly lower than the C_max_ under the reference drug (*p* = 0.0009), meaning that metformin had a higher average maximum plasma concentration in subjects taking metformin plus icosapent ethyl. In contrast to the results observed for AUC_last_ under the fed condition, however, the LSM for C_max_ under metformin eicosapentaenoate was slightly higher than for the reference drugs when taken with a meal. This difference was not statistically significant.

When comparing the PK parameters under the different conditions within each drug treatment group, there were no differences in either parameter for metformin eicosapentaenoate, meaning that the PK profiles defined by AUC_last_ and C_max_ were not different when the drug was taken with or without food. For the metformin plus icosapent ethyl group, on the other hand, both AUC_last_ and C_max_ were significantly higher when taking the drugs while fasting (*p* = 0.0414 and *p* = 0.0164, respectively).

## 4. Discussion

The primary findings from this study are that metformin has lower plasma availability when administered via metformin eicosapentaenoate than when given separately with icosapent ethyl under fasting conditions and that the availability of metformin is not different between the two delivery methods following a meal.

The hope was that AUC_last_ and C_max_ for the metformin PK curves would be larger for metformin eicosapentaenoate than for metformin plus icosapent ethyl under both study conditions, mirroring the greater bioavailability of metformin when esterified to glycine compared to metformin hydrochloride [8] [9]. Had metformin delivered through metformin eicosapentaenoate been more bioavailable, it might have resulted in a greater percentage of the metformin being absorbed and higher PK curves. Since metformin is only 60% absorbed and the unabsorbed portion of the metformin is thought to alter the gut microbiome inducing the gastrointestinal side effects of metformin, better absorption of metformin would be expected to improve the side effect profile associated with the drug [10] [11]. Due to the unanticipated results of this pharmacokinetic study, it appears that metformin eicosapentaenoate has limited advantages over giving the metformin and icosapent ethyl alone. In addition to a lack of improvement in pharmacokinetics, metformin eicosapentaenoate increases the number of required pills from two to four per dose.

One major weakness of this study was that the terminal phase of the metformin PK profile was not sufficiently long to estimate the elimination rate constant k_e_. As a consequence, other PK parameters that are functions of k_e_ could not be estimated. These include half-life (t_1/2_), clearance (Cl), volume of distribution (Vd/F), and area under the plasma concentration curve extrapolated to infinity (AUC_0–∞_). Estimates of these parameters would have allowed for a more comprehensive description and understanding of the metformin PK profile for metformin eicosapentaenoate and subsequent comparison with the reference drugs. On the other hand, an important conclusion is drawn from the analyses just utilizing parameters AUC_last_ and C_max_, which is that the single drug containing metformin and EPA does not appear to improve the availability of metformin in the blood. Metformin eicosapentaenoate actually seems to result in less availability of metformin when taken while in a fasted state.

Based on the results and conclusions, if a similar future study is to be conducted, the number of blood draws following drug administration should be increased in a manner that provides a sufficient number of recorded plasma concentrations in the terminal elimination phase to allow for estimation of all PK parameters. Such a trial is unlikely to be done, since there seems to be little advantage gained from metformin eicosapentaenoate in terms of the pharmacokinetics of metformin.

## Figures and Tables

**Table 1:** Summary statistics of metformin concentrations.

Group^a^	Time (hr)	N	Mean (ng/mL)	SD (ng/mL)	Min (ng/mL)	Median (ng/mL)	Max (ng/mL)
ME Fasting	0	10	0	0	0	0	0
	0.5	10	47.1	50.3	0	34.9	138.0
	1	10	194.8	93.2	36.7	199.5	356.0
	2	10	552.4	184.7	174.0	566.0	874.0
	4	10	723.7	133.5	557.0	692.5	916.0
	8	10	290.5	71.3	187.0	300.5	391.0
	12	10	121.1	37.1	66.3	130.0	164.0

ME Fed	0	8	0	0	0	0	0
	0.5	8	29.1	62.8	0	0	182.0
	1	8	140.2	161.1	0	68.0	368.0
	2	8	412.9	242.4	44.4	434.0	728.0
	4	8	808.5	175.4	518.0	853.0	970.0
	8	8	417.8	113.8	238.0	418.5	569.0
	12	8	164.0	44.8	98.1	165.5	234.0

M + IE Fasting	0	6	0	0	0	0	0
	0.5	6	425.3	158.8	132.0	456.5	574.0
	1	6	785.0	216.0	499.0	754.0	1170.0
	2	6	1077.3	192.8	765.0	1095.0	1350.0
	4	6	949.3	380.7	497.0	881.5	1630.0
	8	6	341.3	165.5	183.0	300.0	615.0
	12	6	145.0	63.9	71.9	129.5	248.0

M + IE Fed	0	6	0	0	0	0	0
	0.5	6	148.6	106.8	37.1	120.1	329.0
	1	6	337.0	196.7	123.0	276.0	594.0
	2	6	613.2	129.5	486.0	567.5	815.0
	4	6	769.5	138.3	578.0	754.5	949.0
	8	6	339.2	63.0	253.0	356.5	410.0
	12	6	132.4	42.4	91.6	118.0	188.0

a ME = metformin eicosapentaenoate, M + IE = metformin plus icosapent ethyl.

**Table 2:** Summary statistics of non-compartmental pharmacokinetic parameters.

Group^a^	Parameter	N	Mean	SD	Min	Median	Max
ME Fasting	Cmax (ng/mL)	10	743.6	140.1	557.0	715.5	916.0
	Tmax (hr)	10	3.8	0.6	2.1	4.0	4.0
	AUC (hr*ng/mL)	10	4573.1	778.1	3208.0	4396.2	5634.5

ME Fed	Cmax (ng/mL)	8	711.3	170.2	540.0	583.0	970.0
	Tmax (hr)	8	3.8	0.7	2.0	4.0	4.1
	AUC (hr*ng/mL)	8	5163.0	842.3	3879.2	5250.7	5187.1

M + IE Fasting	Cmax (ng/mL)	6	1134.0	284.7	765.0	1120.0	1630.0
	Tmax (hr)	6	2.2	1.0	1.0	2.0	4.0
	AUC (hr*ng/mL)	6	6925.1	2191.6	4320.1	6771.2	10655.3

M + IE Fed	Cmax (ng/mL)	6	769.5	138.3	578.0	754.5	949.0
	Tmax (hr)	6	4.0	0.0	4.0	4.0	4.1
	AUC (hr*ng/mL)	6	5179.9	739.2	4334.9	5227.5	6158.2

a ME = metformin eicosapentaenoate, M + IE = metformin plus icosapent ethyl.

**Table 3:** Results of hypothesis tests comparing non-compartmental pharmacokinetic parameters.

Outcome	Test^a^	Reference^a^	Ratio	90% LCL	90% UCL	p-value
AUC (hr*ng/mL)	ME Fasting	M + IE Fasting	67.8	56.8	81.0	0.0010
	ME Fed	M + IE Fed	99.3	82.3	119.7	0.9483
	ME Fasting	ME Fed	88.5	74.9	104.5	0.2175
	M + IE Fasting	M + IE Fed	129.5	105.8	158.6	0.0414

Cmax (ng/mL)	ME Fasting	M + IE Fasting	66.2	54.8	79.8	0.0009
	ME Fed	M + IE Fed	104.4	85.7	127.1	0.7148
	ME Fasting	ME Fed	92.4	75.9	112.4	0.4879
	M + IE Fasting	M + IE Fed	145.7	114.3	185.6	0.0164

a ME = metformin eicosapentaenoate, M + IE = metformin plus icosapent ethyl.

## References

[1] Inzucchi SE, Bergenstal RM, Buse JB (2015). Management of Hyperglycaemia in Type 2 Diabetes, 2015: A Patient-Centered Approach. Update to a Position Statement of the American Diabetes Association (ADA) and the European Association for the Study of Diabetes (EASD). Diabetes Care.

[2] National Institutes of Health, National Institute of Diabetes and Digestive and Kidney Diseases (2013). Diabetes, Heart Disease, and Stroke.

[3] Hu FB, Bronner L, Willett WC (2002). Fish and Omega-3 Fatty Acid Intake and Risk of Coronary Heart Disease in Women. JAMA.

[4] Gillum RF, Mussolino ME, Madans JH (1996). The Relationship between Fish Consumption and Stroke Incidence: The NHANES Epidemiologic Follow-Up Study (National Health and Nutrition Examination Survey). Archives of Internal Medicine.

[5] Kris-Etherton PM, Harris WS, Appel LJ, for the Nutrition Committee (2002). AHA Scientific Statement: Fish Condumption, Fish Oil, Omega-3 Fatty Acids and Cardiovascular Disease. Circulation.

[6] (2012). Vascepa (Icosapen Ethyl) Package Insert.

[7] Gonzalez-Ortiz M, Martinez-Abundis E, Robles-Cervantes JA, Ramos-Zavala MG, Barrera-Druan C, Gonzalez-Canudas J (2012). Effect of Metformin Glycinate on Glycated Hemoglobin A1c Concentration and Insulin Sensitivity in Drug-Naïve Adult Patients with Type 2 Diabetes Mellitus. Diabetes Technology and Therapeutics.

[8] Garza-Ocañas L, Tamez-de la OE, Lujan-Rangel R, Iglesias-Chiesa J, González-Canudas J, Rivas-Ruiz R (2011). Bioavailability of Metformin Glycinate in Healthy Mexican Volunteers: An Open-Label, Single-Dose Clinical Trial. Journal of Diabetes.

[9] Garza-Ocañas L, Tamez-de la OE, Iglesias-Chiesa J, González-Canudas J, Rivas-Ruiz R (2009). Pharmacokinetics and Gastrointestinal Tolerability of DMMET 01 (Glycinate of Metformin): Results of a Prospective Randomized Trial in Healthy Volunteers. Diabetes.

[10] Greenway F, Wang S, Heiman M (2014). A Novel Cobiotic Containing a Prebiotic and an Antioxidant Agments the Glucose Control and Gastrointestinal Tolerability of Metformin: A Case Report. Beneficial Microbes.

[11] Burton JH, Johnson M, Johnson J, Hsia DS, Greenway FL, Heiman ML (2015). Addition of a Gastrointestinal Microbiome Modulator to Metformin Improves Metformin Tolerance and Fasting Glucose Levels. Journal of Diabetes Science and Technology.

